# A novel modified McLaughlin surgery for treating locked chronic posterior shoulder dislocation

**DOI:** 10.1186/s12891-023-06221-3

**Published:** 2023-02-11

**Authors:** Fei Xiong, Qin Yin, Jian Wang, Changbao Wei, Sanjun Gu, Yu Liu

**Affiliations:** 1 Department of Orthopaedics, Wuxi 9Th People’s Hospital Affiliated to Soochow University, NO.999 Liangxi Road, Wuxi, China; 2 Orthopaedic Institute, Medical College, Soochow University, NO.178 Ganjiang East Road, Suzhou, China

**Keywords:** Chronic injury, Posterior shoulder dislocation, Reverse Hill-Sachs lesion, Humeral head fracture

## Abstract

**Background:**

Posterior shoulder dislocation is an uncommon orthopaedics injury and is frequently missed or misdiagnosed, accounting for 2%-4% of all shoulder dislocations, and is associated with the reverse Hill-Sachs lesion. Once posterior shoulder dislocation develops into a chronic disease, it will bring a lot of trouble to the treatment, especially in repairing the humeral defects. Surgical strategies are also developing and innovating to deal with this injury, including transfer of subscapularis tendon or lesser tubercle, humeral rotational osteotomy, autogenous bone graft or allograft. Shoulder replacement seems to be the ultimate and only option when the injury becomes irreparable, although some studies have shown unsatisfactory follow-up results. Considering no gold-standard treatment for locked posterior shoulder dislocation, we described a novel modified McLaughlin procedure for locked chronic posterior shoulder dislocation and evaluated the functional outcomes.

**Methods:**

This study included five locked chronic posterior shoulder dislocation patients with an associated reverse Hill-Sachs lesion, in which the compression surface covered 30–40% of the humeral head. The mean period from injury to receiving surgery was 11.6 weeks (6–24 weeks). All five patients underwent the modified McLaughlin procedure, mainly divided into three steps, including open reduction, transfer of the partial lesser tuberosity and artificial bone to repair the reverse Hill-Sachs defects. The kernel technique was to fix the transferred tuberosity with two lag screws and strengthen it with two Ethibond sutures. The Constant-Murley score (CMS), the range of shoulder motion and the complications were recorded to assess and compare the functional situation of the shoulder postoperatively and postoperatively.

**Results:**

After an average of 19.8 months (12–30) of follow-up, the mean CMS improved to 85.8 ± 4.9 (79–91) compared with 46.0 ± 4.5 (40–52) preoperatively, which showed a significant difference (*p* = 0.001). In the final follow-up, all five patients showed no symptoms of shoulder instability, and there was no pain or limited activity in daily life, thus all patients were satisfied with the results.

**Conclusion:**

Repairing the reverse Hill-Sachs lesion by transferring the partial lesser tuberosity combined with artificial bone fixed by lag screws and sutures can ensure shoulder stability and provide pain relief and good function in patients with locked chronic posterior shoulder dislocation associated with the humeral head defect.

## Introduction

Posterior shoulder dislocation is rare and only accounts for 2%-4% of all shoulder dislocations [1, 2], which can lead to impression fractures on the anterior surface of the humeral head. As reported [3, 4], 40–90% of patients with posterior dislocation of the shoulder suffered from this so-called reverse Hill-Sachs lesion, which may cause significant clinical symptoms and may increase the risk for re-dislocation, progressive joint destruction, and early osteoarthritis [2, 5]. The causes of this injury include electric shock, epileptic seizures or high-energy trauma [6, 7], which is also a challenge for orthopaedic surgeons. The diagnosis is often missed in the acute stage due to inadequate imaging or physical examination, unlike the high accuracy of Dugas sign in anterior dislocation [8–10]. Additionally, in terms of the limitation of shoulder function, some patients may only show external rotation dysfunction and abnormality on the X-ray of axial and standard anteroposterior views [11, 12].

If untreated, reverse Hill-Sachs lesion can present a risk for persistent posterior glenohumeral instability, similar to anterior shoulder instability, while deciding on the appropriate treatment depends on the time from injury, associated fracture presence, lesion size, current functions and patients' expectations [6, 7, 13–15]. Current surgical interventions addressing the anterior humeral head defect can be divided into anatomical and non-anatomical reconstructions. The anatomical procedures aim to restore the original shape of the humeral head, including open reduction or filling of the humeral head defect with either autograft or allograft [16, 17]; by distinction, the non-anatomical technique attempt to restore stability by filling the defect with the subscapularis tendon [18].

McLaughlin [19] was the first to describe the “tenodesis” of the transportation of the subscapularis tendon into the bone defects. Subscapularis tendon served as a dynamic stabiliser that resists posterior translation [20], and advantageous of McLaughlin Procedure was proved in maintaining the most function of the subscapularis tendon. Since then, several procedures have shown reliable clinical results to solve this problem [5, 13, 21–24]. This technical note describes a modified McLaughlin procedure for treating reverse Hill-Sachs lesion mainly through the open reduction and “tenodesis” of the partial lesser tuberosity with subscapularis tendon into the Hill-Sachs lesion using lag screws and two Ethibond sutures. The rationale of this procedure was to provide more secure fixation for the subscapularis tendon and ensure the part function of the subscapularis and the integrity of the bicipital groove as much as possible.

## Methods

### Patients

This prospective single-centre study reported a consecutive case series of patients who underwent the modified McLaughlin procedure to treat chronic posterior dislocation of the shoulder with humeral head defects, between April 2016 and Jan 2018. We defined a chronic dislocation as a time from injury > 6 weeks.

**Fig. 1:**
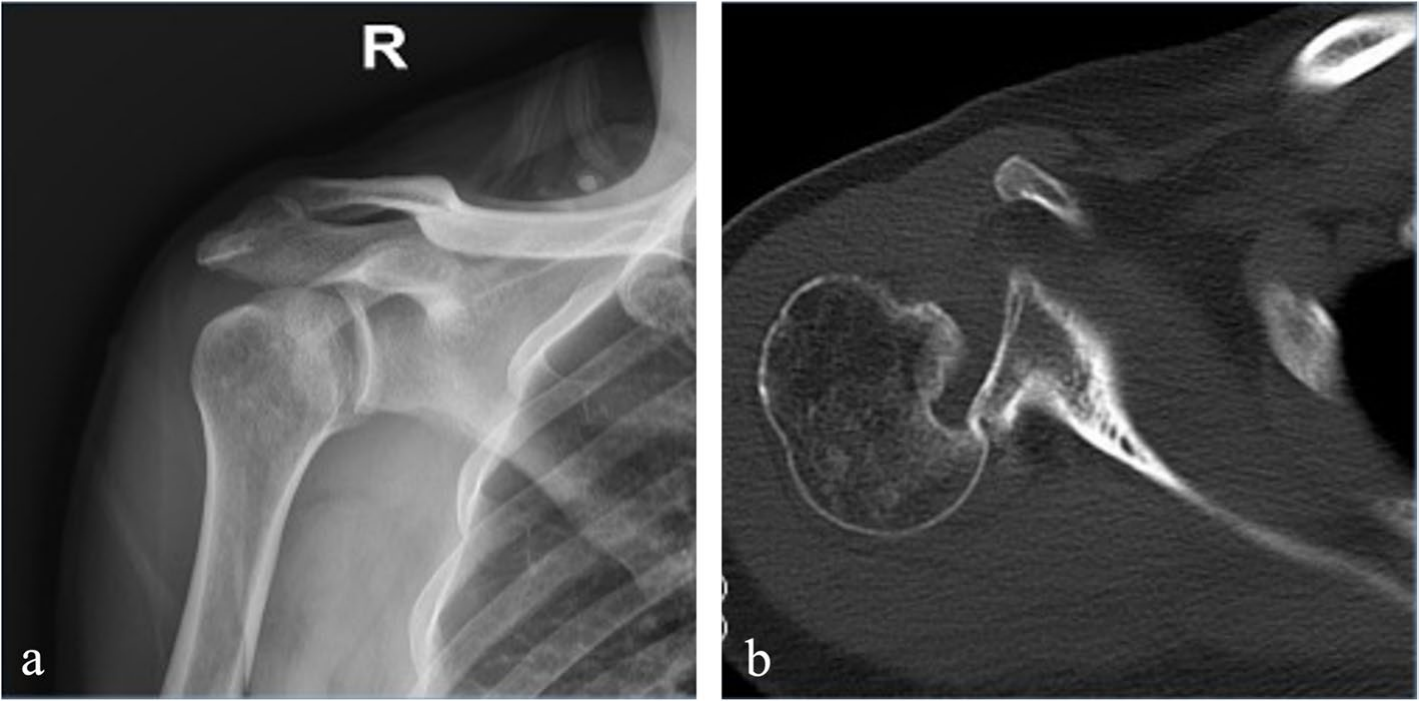
Preoperative radiographs of the right shoulder of a patient with a 8-week delay from injury to diagnosis showing a chronic locked posterior dislocation of the shoulder with a large reverse Hill-Sachs lesion

Preoperatively and postoperatively, all patients underwent clinical and imaging examinations that included radiographs (true anteroposterior, scapular Y, and axillary views) and computed tomography (CT)(Fig. 1). The range of motion, CMS, and VAS were assessed preoperatively and at the follow-up by the doctor responsible for this operation. The decision to perform the modified McLaughlin procedure was made in cases with chronic posterior dislocation of the shoulder and a reverse Hill-Sachs defect involving < 40% of the humeral head as determined by the calculation method in the preoperative axial CT scan reported by Gerber et al. [25]. Patients with surgical neck fractures in the humerus or the tuberosities or cases undergoing shoulder arthroplasty were excluded. Subsequently, treatment options, which include non-operative and operative approaches, should be discussed with the patient. All patients willing to undergo operation will sign an informed consent form.

### Operative procedure

**Fig. 2:**
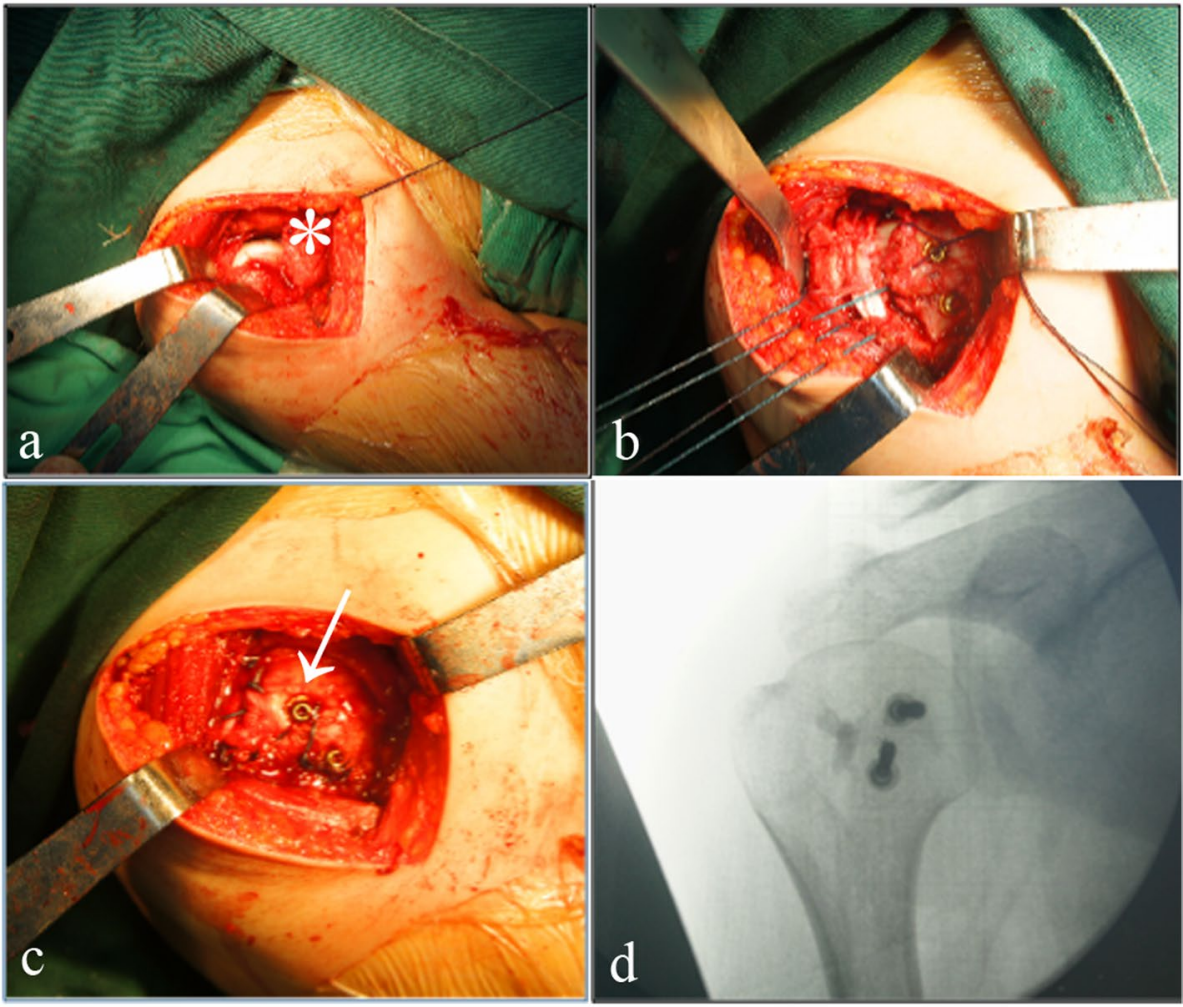
**a** Intraoperative photograph after humeral reduction showing a large reverse Hill-Sachs lesion of the right shoulder. **b**, **c** Fixation of the transferred tuberosity with two lag screws and strengthen it with two Ethibond sutures. **d** Immediate intraoperative fluoroscopy scans showing the results of the modified McLaughlin procedure. The star indicated the reverse Hill-Sachs defect lesion. The arrow indicated the fixed reverse Hill-Sachs defect lesion

All surgical procedures were performed by the corresponding author. The operation was performed with the patient under general anaesthesia combined with an interscalene block and placed in the beach chair position. The deltopectoral approach was chosen, in which the skin incision starts proximal to the coracoid and extends distally over the deltopectoral interval, which further facilitates exposure of the proximal humerus. The arm was subsequently externally rotated to further adequately expose the borders of the subscapularis and reduction (Fig. 2a). The osteotomy was started from the distal end of the lesser tuberosity, which generally did not exceed half of the lesser tuberosity. Before one attempts a reduction, adhesions between the posterior glenoid and humeral head should be released, including the attached subscapularis tendon. Reduction of the dislocation is achieved with lateral distraction and external rotation; meanwhile, care should be taken to avoid further damage to the humeral head and glenoid. After reduction, the size of the head defect was then evaluated. If considered viable, the modified McLaughlin procedure is performed. Freshness was performed using curettes to prepare the breeding ground for the lesser tuberosity. Tunnels for the sutures were drilled in the remaining lesser tuberosity using 2 mm Kirschner wires. The lesser tuberosity was then transferred to the head defect, and the artificial bone (WRIGHT, USA) was implanted in the interval. Fixation of the lesser tuberosity was performed using two 4.0 mm lag screws (AO, Switzerland), and 2–4 Ethibond sutures (size 2) were applied to strengthen the fixation in a trans-osseous fashion (Fig. 2b; c). Intraoperatively, the stability of the shoulder and the transferred tuberosity were assessed by direct observation through the entire range of motion of the shoulder. Finally, the position of shoulder joint was confirmed by intraoperative fluoroscopy (Fig. 2d). The mean operative time was 75 min.

### Postoperative management

Postoperative antibiotic prophylaxis with a first-generation cephalosporin was administered within 24 h. For four weeks, the shoulder was immobilised in a neutral position using shoulder support. Internal or external rotation movements were not allowed, while wrist and hand exercises were instructed and encouraged. After the first period, a progressive rehabilitation physical and occupational therapy program was initiated, including a passive range of motion of external rotation and forward flexion, supplemented with an acceptable active range of motion. Total activity and a full range of motion were allowed at three months postoperatively. The transferred lesser tuberosity within the reverse Hill-Sachs defect was evaluated using plain radiographs and CT scans postoperatively and in the third-month follow-up.

### Statistical methods

Data were expressed using mean and standard deviation (SD). Preoperative and postoperative range of motion and functional scores were analysed by the paired *t* test. *p* < 0.05 was considered significant. Analyses were conducted using SPSS Statistics (SPSS for Windows, version 17.0, Inc, Chicago IL).

## Results

**Fig. 3:**
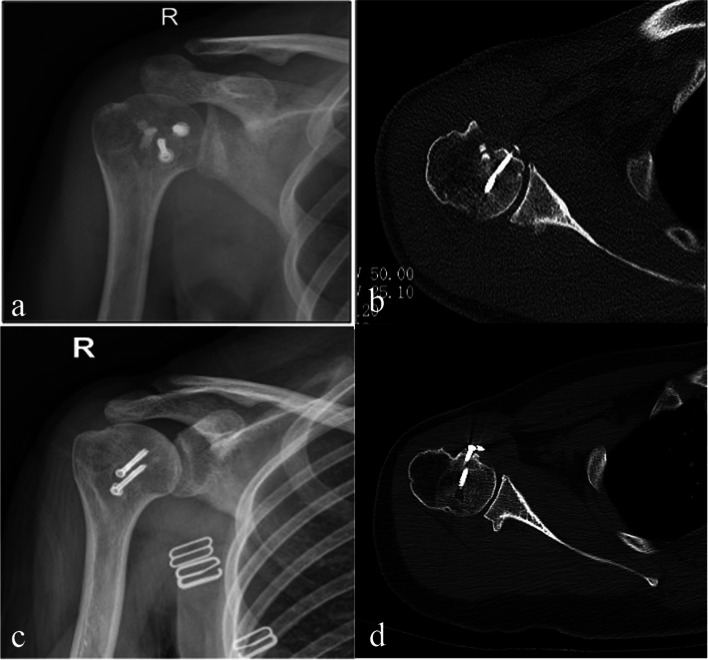
**a**, **b** The fixation of the transferred lesser tuberosity within the reverse Hill-Sachs defect was evaluated using plain radiographs and CT postoperatively. **c**, **d** The presence of the bicipital groove, the absorption of artificial bone, and the healing of the reverse Hill-Sachs defect lesion were evaluated using plain radiographs and CT in the third-month follow-up

**Fig. 4:**
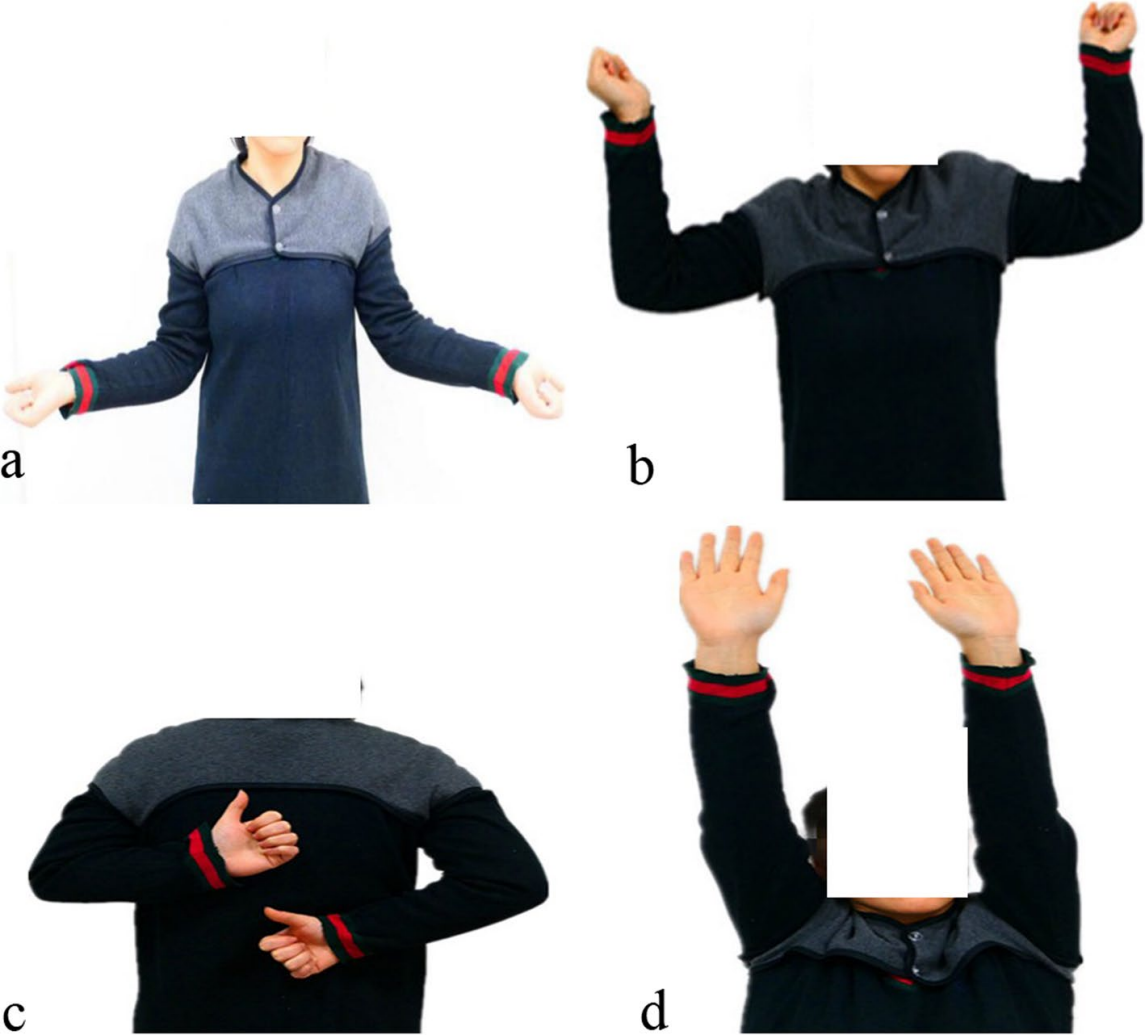
Good functional results of the right shoulder were observed 1 years after surgery

Between April 2016 and Jan 2018, the modified McLaughlin procedure was performed on five patients whose main mechanism of injury for dislocation were electric shock and falling injury. The five patients included two male and three female, and the mean age was 51 years (27–81). The time between dislocation and surgery ranged from six to 24 weeks, with a mean of 11.6 weeks (Table 1). The mean size of the reverse Hill-Sachs defect was 36.2% (30%- 40%). The mean follow-up period for clinical data was 19.8 months (12–30 months). Postoperative radiographs and CT scans confirmed the proper screws fixation and artificial bone graft (Fig. 3a; b). In the third-month follow-up, imaging confirmed the presence of the bicipital groove, the absorption of artificial bone, and the healing of defects (Fig. 3c; d). Range of motion improved significantly after surgery: Mean forward flexion improved from 71.00 ± 8.94 to 152.00 ± 13.51 (*p* < 0.001), mean external rotation improved from 4.00 ± 5.48 to 58.00 ± 5.70 (*p* < 0.001), mean abduction rotation improved from 50.00 ± 11.73 to 148.00 ± 19.24 (*p* < 0.001), and mean internal rotation improved from buttock region ± 1 vertebral level to L2 ± 2 vertebral levels (T12—L4) (Fig. 4). CMS was used to assess the functional situation preoperatively and postoperatively, and the outcome measures demonstrated significant improvements. The mean CMS improved from 46.00 ± 4.47 to 86.20 ± 5.36 (*P* < 0.001) (Table 2). The mean VAS (visual analog scale) improved from 46.00 ± 4.47 to 86.20 ± 5.36 (*P* < 0.001) (Table 2). Postoperatively, all of these five patients were satisfied with the final results, and none of them complained of pain or obvious restricted daily living activities. There was no case of shoulder instability, avascular necrosis, recurrent dislocation, infection, or neurologic injury.

**Table 1:** Patient Data

Patient no	Age, yr	Gender	Cause	Time to Surgery, weeks	Defect size, %	Follow-up, weeks	Preoperative ROM and functional scores						Postoperative ROM and functional scores					
							FF	ER°	AR	IR	CMS	VAS score	FF	ER	AR	IR	CMS	VAS score
1	36	male	fall	24	30	12	80	10	50	Buttock	48	6	165	65	170	L3	92	2
2	40	female	seizure	8	40	15	70	0	40	L5	46	5	155	60	140	T10	84	2
3	81	female	fall	6	40	18	80	0	70	Buttock	52	2	130	50	120	L2	79	1
4	71	female	fall	11	35	30	60	10	45	L5	44	4	150	55	150	L2	85	1
5	27	male	seizure	8	30	24	65	0	45	Lateral thigh	40	6	160	60	160	T12	91	0

*ROM* Range of motion, *FF* Forward flexion, *ER* External rotation, *AR* Abduction rotation, *IR* Internal rotation, *CMS* Constant-Murley score, *VAS* Visual analog scale

**Table 2:** Preoperative and postoperative range of motion and functional results

	Preoperative	Postoperative	*p* value
FF	71.00 ± 8.94	152.00 ± 13.51	0.0005
ER	4.00 ± 5.48	58.00 ± 5.70	< 0.0001
AR	50.00 ± 11.73	148.00 ± 19.24	0.0014
IR	Buttock ± 1 vertebral level	L2 ± 2 vertebral level	NA
CMS	46.00 ± 4.47	86.20 ± 5.36	0.0005
VAS score	4.60 ± 1.67	1.20 ± 0.84	0.0139

*FF* Forward flexion, *ER* External rotation, *AR* Abduction rotation, *IR* Internal rotation, *CMS* Constant-Murley score, *VAS* Visual analog scale; Data are presented as mean ± standard deviation

## Discussion

The main finding of our study was the modified McLaughlin procedure could mitigate the locked chronic posterior shoulder dislocation, especially in improving CMS, which improved from 46.00 ± 4.47 to 86.20 ± 5.36 (*P* < 0.001). In addition, osteotomy of partial lesser tuberosity ensured the integrity of the bicipital groove as much as possible. Meanwhile, the integrity of the subscapularis could be preserved as much as possible through partial osteotomy so that patients can obtain good internal rotation function postoperatively. The fixation method using lag screws and Ethibond sutures also effectively improved the firmness.

Posterior shoulder dislocation is an uncommon injury with the associated compression fracture of the humeral head, also known as reverse Hill-Sachs lesion. The patient's main complaints are usually pain and limited external rotation of the shoulder. Misdiagnosis and missed diagnoses often occur due to a lack of a highly specific physical examination method, unlike Dugas sign in anterior dislocation [8–10]. The findings of anteroposterior imaging can only provide limited evidence; although the axillary position can provide more information, it is limited by the forced position caused by pain. In our series of cases, one patient aged 81 came to the hospital only because of limited external rotation, had no pain symptoms and could still do housework after injury, whose diagnosis was finally confirmed according to the CT scan. Some reports demonstrated that the elderly may be more tolerant of the pain caused by posterior shoulder dislocation and can even meet the needs of daily life through the compensation function from anterior curvature, even if the compressed humeral head and scapula glenoid rim formed pseudo joints [18, 26]. All the above problems may cause delayed diagnosis and treatment, which can have serious deleterious effects on shoulder function.

The treatment strategy for posterior shoulder dislocation depends on the size of the humeral head defect, duration of the dislocation, age and functional expectations of patients [6, 7, 13–15]. Taking 25% of head defects as the cut-off point, as several studies showed [6, 7, 13–15], less than which the closed reduction of the defect may be attempted. For defects between 25 and 40% of the articular surface, the reconstruction with a graft was needed, although challenging, to stabilise the shoulder and avoid progressive joint destruction and early osteoarthritis. When the bone lesion developed to more than 40%, hemi or total shoulder arthroplasty seemed to be the last salvage procedure [27, 28], while the clinical results were not satisfactory in some researches [29, 30], especially in one of which 5 of 12 patients complained the results were unacceptable[30].

Reconstruction of the compression humeral head is the emphasis and difficulty of the surgical procedure, and various surgical approaches have been described to address humeral bone loss historically. McLaughlin [31] first reported a treatment option for chronic posterior shoulder instability cases associated with 20–50% of the head surface defect. Subscapularis tendon was recommended to transfer into the defect to separate the lesion and the glenoid rim, and the engagement was prevented, by which recurrent dislocations and early degenerative changes were avoided. Nevertheless, the concern when applying this strategy was whether the transferred tendon and impacted bone could heal as expected. This procedure was later modified by Hawkins et al. [28], who performed a lesser tuberosity osteotomy, then transferred the lesser tuberosity with the attached subscapularis tendon and filled it in the defect using screws for fixation. Other studies also described proximal humerus rotational osteotomy [32, 33], the elevation of the depressed articular surface and autogenous bone or allograft bone [2, 16, 34], and Krackhardt et al. [35] first introduced an arthroscopic technique.

The most important finding of this study was that the presented modified McLaughlin procedure was an easily reproducible approach for applicability to reverse Hill-Sachs lesions. This technique mainly included open reduction, transfer of the partial lesser tuberosity and artificial bone implant, in which the transferred tuberosity was fixed by two lag screws and then strengthened by 2–4 Ethibond sutures. The transferred lesser tuberosity ensured the integrity of the humeral tubercle, which reduced the risk of pain caused by the slipping of the biceps brachii tendon, while the attached subscapularis tendon increased the healing opportunities, like a vascularised bone flap. Because the proximal humerus was mainly composed of cancellous bones, fixation by screws alone was unreliable, while applying the Ethibond suture and trans-osseous technique made early rehabilitation possible. Furthermore, the artificial bone was used to repair the defect; by doing so, the re-collapse of the articular surface was prevented.

Our results demonstrated that the modified McLaughlin operation was effective, and the results were good within 1–3 years of follow-up. Although the CMS of the 81-year-old patient mentioned above only increased from 52 to 79 postoperatively, she still thanked for the satisfactory results, which may be due to the improvement of joint activity and low preoperative expectation. During the operation, a large rotator cuff tear was confirmed; through reflection, shoulder arthroplasty may be a better choice. Nevertheless, there were no severe complications such as severe pain, arthritis and humeral head collapse at the last follow-up.

Only a few studies have described the treatment of chronic posterior locked shoulder dislocation, mostly with a small number of cases, which was also one of the limitations of this study. Nevertheless, the results of the small sample size provided encouraging clinical outcomes based on the curative effect with statistical differences and nonmention of internal fixation failure, non-union of lesser tuberosity transferred and recurrent instability. In addition, the short follow-up time was another limitation, so caution was needed in the promotion and application, which is why it also calls for more research and a longer follow-up time for this strategy.

The surgical procedure in this case series was provided to reconstruct the shoulder joint in a nearly anatomical way by transferring the partial lesser tuberosity with artificial bone implantation, which was safe, simple, effective and reliable. Besides comparable effectiveness among other techniques, this method also has unique advantages, such as eliminating the cost burden of a secondary surgery to remove internal fixation. This manuscript can supply orthopaedic surgeons with a new option who face clinical and surgical decisions about posterior shoulder dislocation associated with a reverse Hill-Sachs lesion patient in daily clinical practice.

## Conclusion

Repairing the reverse Hill-Sachs lesion by transferring the partial lesser tuberosity combined with artificial bone fixed by lag screws and sutures can ensure shoulder stability and provide pain relief and good function in patients with locked chronic posterior shoulder dislocation associated with the humeral head defect.

## Data Availability

All data generated or analyzed during this study are included in this published article.

## Abbreviations

CMS: Constant-Murley score
CT: Computed tomography
SD: Standard deviation
